# The effect of an affect, sensation seeking, and premeditation on risky decision-making: Conditional process analysis

**DOI:** 10.1371/journal.pone.0281324

**Published:** 2023-02-06

**Authors:** Adarsh K. Verma, Usha Chivukula

**Affiliations:** Centre for Health Psychology, School of Medical Sciences, University of Hyderabad, Hyderabad, Telangana, India; Dokkyo Medical University School of Medicine, JAPAN

## Abstract

Risks often accompany available choices in decision-making, particularly where the monetary factor gets involved. Researchers have explored the pathways underlying risky decision-making for decades, but most of these pathways have explored the factors individually rather than through a holistic approach. The present study examines the role of personality, cognitive, and biological components in risky decision-making. Here, the Iowa Gambling Task (IGT) paradigm is used to study the targeted outcome variable (IGT payoff) in 281 healthy students. Two moderation-mediation models hypothesized sensation seeking and lack of premeditation as predictors of IGT payoff. Positive and negative moods prior to IGT administration were considered mediators, and age and gender as moderators in predicting payoff. The hypothesized models were tested using conditional process analysis. Results indicate that both predictors significantly negatively predict payoff while moderated by gender and age categories. Interestingly, the indirect relationships apply to 21–34 years old men and 21–25 years old women. These age and gender-specific findings in apparently healthy participants highlight the need for replicating the current research in different age groups and clinical populations involving maladaptive decision-making patterns.

## Introduction

Decision-making is a complex cognitive process that everyone uses on a day-to-day basis. Nevertheless, this process becomes critical when some potential risk is associated [1]. Evidence suggests that deficits in decision-making are more prominent in gambling disorders than in other conditions [2]. Iowa Gambling Task (IGT) is a widely used paradigm to study impaired decision-making with an anticipatory risk (loss or gain) context. Hence, the IGT paradigm has been widely used in psychological and neurological studies exploring decision-making processes with involved risks [3–5].

IGT studies witnessed the selection of disadvantageous options as the prominent predictor of maladaptive decision-making [6, 7]. Healthy participants gradually learn the pattern of outcomes and exhibit advantageous decisions in the long term compared to the clinical population and poor decision-makers [7]. This hypothesis further helps explore other associated psychological processes that potentially influence decision-making, such as affect, personality, perception, and prospection [5, 8–10].

Previous literature prominently associates sensation-seeking with risky decision-making [11, 12]. Sensation-seeking is a personality characteristic that pushes an individual to seek thrill and excitement in day-to-day activities. Under uncertainty, this characteristic tends to play a dominant role in guiding decisions and individual behaviors. The high sensation-seeking trait impairs the sensitivity to recurring losses or adverse outcomes and degrades performance [12]. Delibas et al. [13] reported the negative impact of high sensation-seeking in clinical patients but also cautioned about interpreting it as a deficit in decision-making.

Premeditation is another cognitive process that is implicated in decision-making. It is a process of conscious planning before performing any activity. It is an under-reported variable in the decision-making literature. The existing studies on premeditation and decision-making show that people with a higher score on premeditation tend to perform better on decision-making tasks [14, 15]. Additionally, it is reported to influence decision-making by modulating the affective process [15]. Engaging in premeditation helps individuals exert control over their actions [16]. On the other hand, lack of premeditation is implicated in maladaptive or disadvantageous decision-making [15].

The studies evaluating the role of affect in this context propose a significant role of affective states in forming decisions [5, 10, 17]. For example, Chung et al. [18] evaluated the role of affect/mood on an economic decision-making task by manipulating the mood state. Findings indicate that both the positive and negative affect significantly influence the decision on financial tasks, where the positive mood was reported to be linked with advantageous decisions [5, 18]. Further, during the repetitive trial in the decision-making task, the emotional changes during the earlier trials tend to influence the later trials’ performance [17].

Additionally, the decision-making ability tends to be age and gender-dependent [19, 20]. Few existing studies that explored gender and difference in context to IGT performance present conflicting evidence [21, 22]. Reavis & Overman [21] reported a significant gender difference in IGT outcomes where men made more advantageous decisions than women. Still, no significant difference was noted between younger (*M* = 28 years) and older (*M =* 69 years) adults. Contrary to the age finding, other studies suggested significant differences across younger and older categories in IGT performance [22, 23]. The literature lacks enough evidence to clearly establish age and gender differences in decision-making involving risk and money.

Hence, the exploration of mediating and moderating mechanisms in decision-making is not emphasized in existing literature in the context of age, gender, affect, sensation-seeking, and premeditation combinedly. The proposed study comprehensively addresses how different age categories (within young adults), gender, and affect play moderating and mediating roles in decision-making. It aims at bridging the gap between existing literature by concurrently evaluating the role of these variables.

### Objective

- To understand how sensation seeking, lack of premeditation, and affective state prior to involvement in a decision-making influence the decision-making process.
- To explore the role of age and gender as moderators in the decision-making process.

### Hypotheses

H_1_: Sensation-seeking and premeditation significantly predict performance on a decision-making task (payoff).
H_2_: Affect prior to the decision-making task significantly mediate the relationship between predictors and outcome variables while being moderated by age and gender.

## Method

### Sample

The study was conducted on 281 college students aged 21 to 34 (*M* = 25.37, *SD* = 3.42). The exclusion criteria for recruiting participants were self-reported visual impairments, tactile impairments, or past insult to the brain. The inclusion criteria was a basic understanding of the English language.

### Design

The current study used an ex-post-facto research design for examining the role of different variables, i.e., age, gender, affect, sensation-seeking, and lack of premeditation on the outcome variable (decision-making). The affect/mood of the participants is not manipulated in the current study. The preexisting mood score before involvement in IGT was used to evaluate its role in decision-making. Hence, it qualifies this study for ex-post-facto design.

### Tools/Measures

#### Modified Iowa Gambling Task (IGT-M)

The Modified Iowa Gambling Task (IGT) is a self-modified simulated card game task for exploring advantageous and disadvantageous decision-making. Based on the original IGT framework, 40 trials were scored [7]. The first ten trials were considered practice trials and were replicated at the end of the 40 trials sequence. Hence, the modified task presents the participant with four differently programmed card decks across 50 trials. Decks C and D with lower rewards in each trial were programmed as advantageous, whereas decks A and B with greater rewards were disadvantageous in the long run. IGT-M measure was scored for the payoff/mean net score (P; the difference between a number of selections from good and bad decks) for the last 40 trials [24, 25].

#### S-UPPS-P impulsive behavior scale

This scale is a shorter version of the original UPPS-P scale that measures multi-faceted impulsivity constructs [26]. It consists of 20 items, divided into five subscales. It consists of a 4-point Likert-type response pattern. The sensation-seeking and lack of premeditation scores were obtained by adding the scores of respective items after reverse coding the specified items. The scale has good psychometric properties, with internal consistency for different subscales ranging from 0.74 to 0.88 [26].

#### Brief Mood Introspection Scale (BMIS)

The BMIS is a self-report measure that captures a person’s affective state at any given moment [27]. It includes 16 adjectives that assess the current mood. It includes a 4-point response scale including "definitely do not feel," "do not feel," "slightly feel," and "definitely feel." The positive and negative mood scores are calculated by adding positive and negative adjectives, respectively.

### Setting

The study is conducted in a laboratory setting to avoid any potential interference of extraneous variables, including noise and lighting.

### Procedure

The study was carried out digitally through the offline mode of communication. The participants were invited using convenient sampling. The study was conducted in a laboratory setting. Initially, the participants were briefed about the study (while withholding the necessary information that could manipulate the results), and written informed consent was obtained from them. Then, they were screened for inclusion and exclusion. It was followed by taking down the demographic and socio-economic details and administering impulsivity and affectivity measures. After a 2-minutes break, the Modified Iowa Gambling Task was administered to obtain the decision-making scores. Based on the earned tokens (instead of real money) in IGT-M, the incentives are provided to the participants with an equivalent amount depending on the participant’s preferences. Thereafter, the participants were given a thorough debriefing and thanked. This present study is conducted in accordance with ethical approval [No. UH/IEC/2022/272] received from the University of Hyderabad Institutional Ethics Committee.

### Statistical analysis

Descriptive and correlational analyses were performed using IBM SPSS v23. For evaluating the mediating and moderating relationships in predicting decision-making outcomes, conditional process analysis was performed using the macro 4.1 SPSS plugin developed by Hayes [28].

## Results

**Fig 1:**
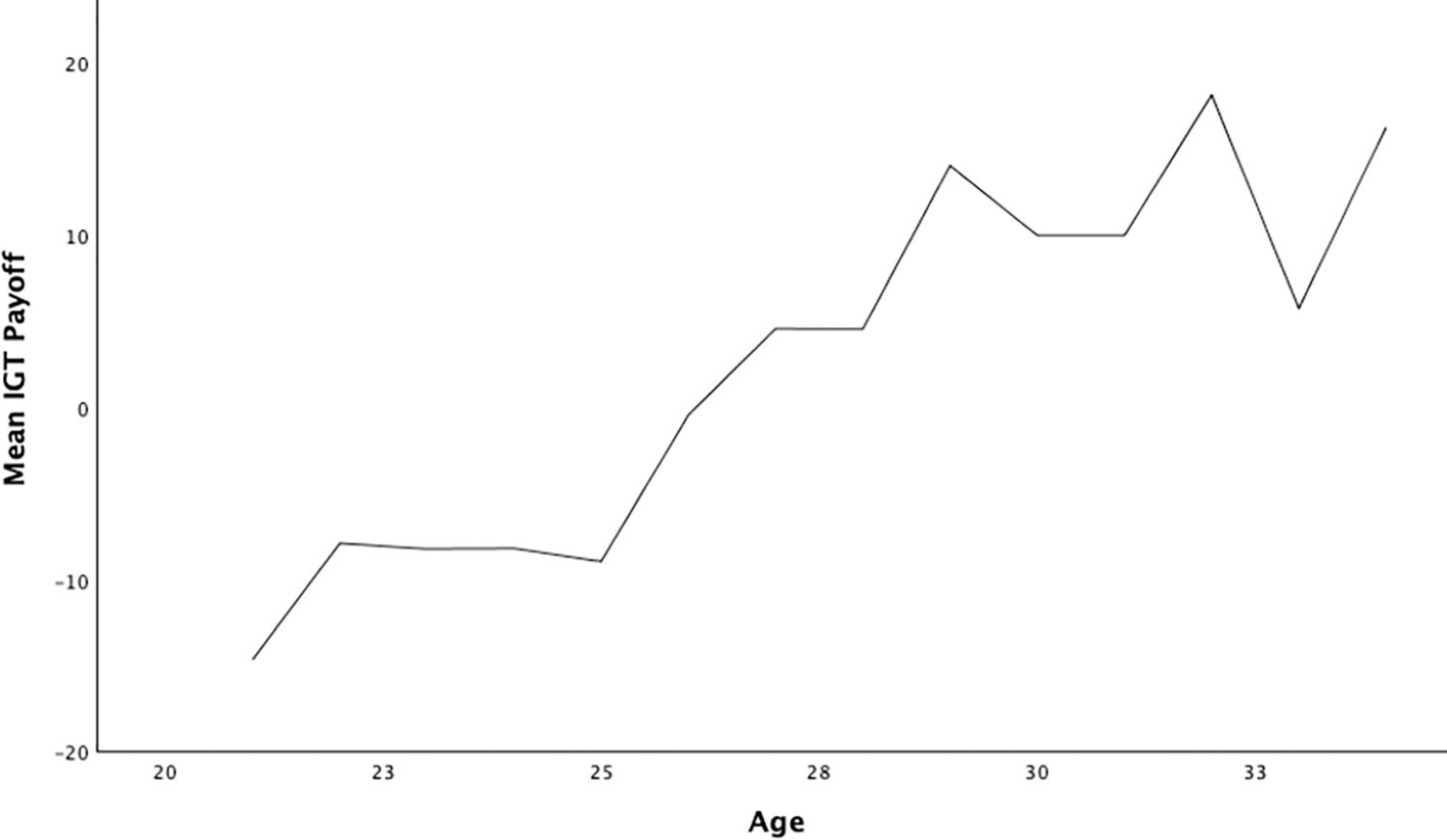
IGT payoff trend over 21–34 years old study sample.

The statistical analysis was performed on the data obtained from 281 participants. In the literature, cognitive abilities are proposed to be significantly influenced by age and peaking around 25 years of age (towards the end of emerging adulthood) [29–31]. Hence, the participants are divided into two age categories, with 25 years as a cut-off to understand the moderating role of age [30, 31]. The two age categories include 21 to 25 years old and 26 to 34 years old. The descriptive and correlational results for different variables are presented in Tables 1 and 2, respectively. The trend of IGT payoff change over 21 to 34 years of age in the study sample is depicted in Fig 1.

**Table 1:** Descriptive statistics.

		21–25 yrs.		26–34 yrs.	
	Gender	M	F	M	F
	n	99	73	65	44
Age	Mean	23.10	22.96	29.37	28.55
	SD	1.12	1.10	2.69	2.03
Sensation Seeking	Mean	13.73	12.12	8.74	8.52
	SD	2.21	3.12	3.70	3.42
Lack of Premeditation	Mean	11.79	10.10	8.03	7.11
	SD	3.16	3.76	3.39	2.79
Positive Mood	Mean	26.97	25.93	17.29	17.66
	SD	3.07	4.65	6.50	6.25
Negative Mood	Mean	13.98	14.21	23.38	22.70
	SD	3.23	4.73	5.99	5.50
Payoff	Mean	-10.08	-6.70	9.08	6.77
	SD	9.81	11.72	12.68	13.29

**Table 2:** Correlation matrix.

	1	2	3	4	5	6	7
1. AgeCat	-						
2. Gender	-0.021	-					
3. Lack of Premeditation	-.441**	-.173**	-				
4. Sensation Seeking	-.571**	-.129*	.677**	-			
5. Positive Mood	-.665**	-0.022	.562**	.675**	-		
6. Negative Mood	.683**	-0.023	-.540**	-.609**	-.820**	-	
7. Payoff	.576**	0.030	-.617**	-.673**	-.727**	.668**	-

** Correlation is significant at the 0.01 level (2-tailed).

* Correlation is significant at the 0.05 level (2-tailed).

**Fig 2:**
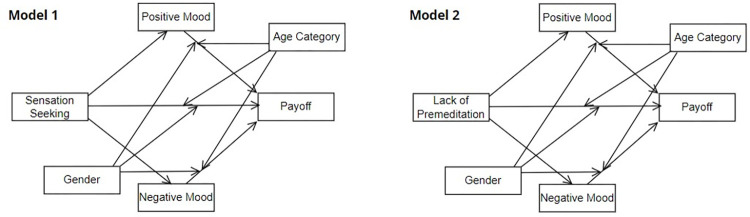
Mediation-moderation models 1 and 2.

The mediation-moderation models 1 and 2 hypothesize premeditation and sensation-seeking as significant predictors of payoff and sensitivity to the frequency in IGT. The positive and negative moods prior to the performance on IGT are considered mediators, and age category and gender are the moderators between mediators and the outcome variable and between predictor and outcome variable (Fig 2, Panel 1, 2). The mediation-moderation models were analyzed using conditional process analysis.

### Model 1

The results from the first mediation-moderation model are presented in Table 3. IGT payoff is directly negatively predicted by sensation-seeking (*b* = -1.21, *p* < .01) and positive mood (*b* = -.86, *p* < .01), whereas positively predicted by negative mood (*b* = .59, *p* < .05). Additionally, gender also predicted the IGT payoff (*b* = 22.97, *p* = .05).

**Table 3:** Conditional process analysis summary for Model 1.

		IGT Payoff (Y)					
						95% BCa CI	
		Estimate	*SE*	*t*	*p-value*	*LL*	*UL*
constant		21.48	9.63	2.23	0.03	2.520	40.448
Sensation Seeking (X)		-1.21	0.38	-3.21	0.00	-1.947	-0.466
Positive Mood (M1)		-0.86	0.26	-3.33	0.00	-1.377	-0.353
Negative Mood (M2)		0.59	0.25	2.33	0.02	0.093	1.093
Age Category		-16.45	11.88	-1.38	0.17	-39.843	6.952
Gender		22.97	11.65	1.97	0.05	0.034	45.913
*θ*_*X→Y*_							
21–25 yrs. age	Men	-1.21	0.38	-3.21	0.00	-1.947	-0.466
	Women	-1.54	0.33	-4.70	0.00	-2.181	-0.893
26–34 yrs. age	Men	-0.68	0.34	-2.00	0.05	-1.340	-0.011
	Women	-1.01	0.38	-2.66	0.01	-1.752	-0.260
*θ*_*X→M1*_ *θ*_*M1→Y*_							
21–25 yrs. age	Men	-1.03	0.36	-	-	-1.758	-0.346
	Women	-1.42	0.35	-	-	-2.118	-0.756
26–34 yrs. age	Men	-0.57	0.36	-	-	-1.270	0.149
	Women	-0.95	0.42	-	-	-1.711	-0.072
*θ*_*X→M2*_ *θ*_*M2→Y*_							
21–25 yrs. age	Men	-0.62	0.30	-	-	-1.204	-0.023
	Women	0.13	0.30	-	-	-0.405	0.773
26–34 yrs. age	Men	-0.81	0.33	-	-	-1.411	-0.116
	Women	-0.07	0.38	-	-	-0.775	0.746

*Note*. 95% BCa CI = Bias-corrected and accelerated bootstrap confidence interval (CI) based on 10,000 bootstrap resamples; LL = lower limit, UL = upper limit.

**Fig 3:**
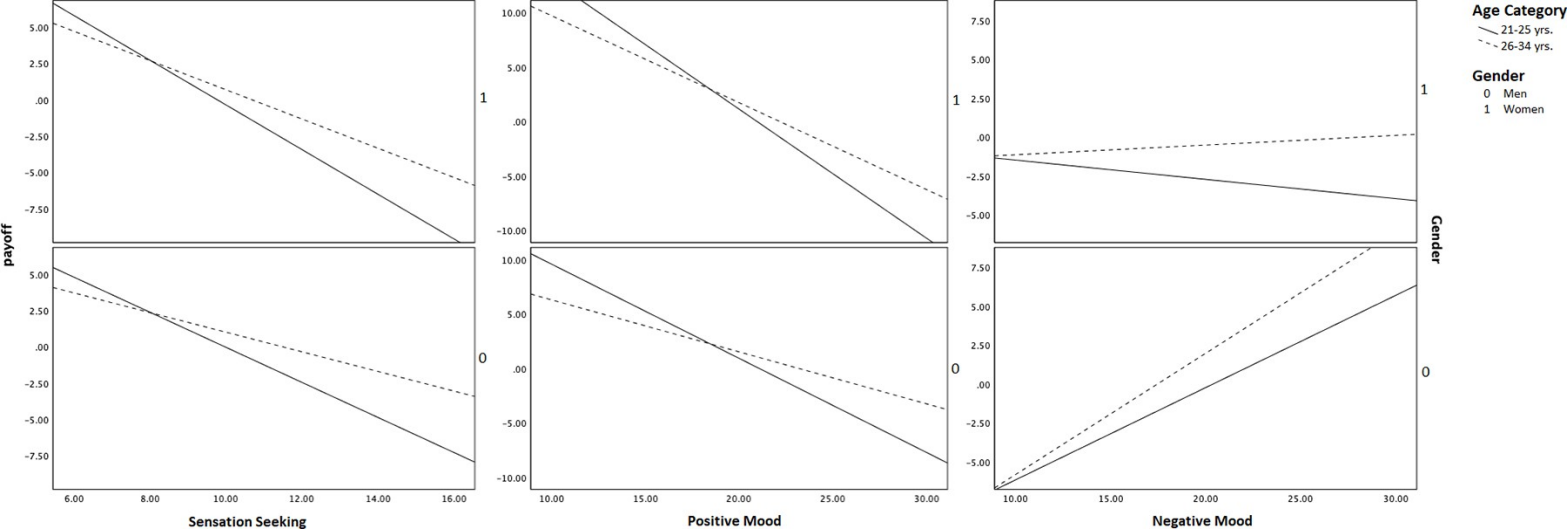
Conditional relationship in mediation-moderation model 1.

Conditional direct effects suggest that both age categories in both genders, i.e., men and women, significantly negatively moderate the direct relationship between sensation-seeking and payoff (Table 3). The conditional indirect effect through positive mood (θ_X→M1_θ_M1→Y_) is significantly moderated by both the genders in the 21–25 years age category (men, *b* = -1.03, 95% BCa CI [-1.758, -.346]; women, *b* = -1.42, 95% BCa CI [-2.118, -.756]), but only by women gender in 26–34 years age category (*b* = -.95, 95% BCa CI [-1.711, -.072]). The conditional indirect effect through negative mood (θ_X→M2_θ_M2→Y_) is significantly moderated by both the age categories, but only in men (Table 3). The visual depiction of moderating effect is presented in Fig 3.

### Model 2

In the second model, premeditation is considered the predictor of payoff in IGT while keeping mediating and moderating variables the same as in Model 1. Here, payoff significantly directly predicted lack premeditation (*b* = -1.00, *p* < .01), positive mood (*b* = -.94, *p* < .01) (Table 4).

**Table 4:** Conditional process analysis summary for Model 2.

		IGT Payoff (Y)					
						95% BCa CI	
		Estimate	*SE*	*t*	*p-value*	*LL*	*UL*
constant		20.53	9.52	2.16	0.03	1.776	39.276
Lack of Premeditation (X)		-1.00	0.30	-3.32	0.00	-1.585	-0.406
Positive Mood (M1)		-0.94	0.25	-3.79	0.00	-1.429	-0.452
Negative Mood (M2)		0.47	0.26	1.79	0.07	-0.047	0.984
Age Category		-14.80	11.78	-1.26	0.21	-37.983	8.388
Gender		21.09	11.70	1.80	0.07	-1.951	44.129
*θ*_*X→Y*_							
21–25 yrs. age	Men	-1.00	0.30	-3.32	0.00	-1.585	-0.406
	Women	-1.42	0.27	-5.20	0.00	-1.964	-0.885
26–34 yrs. age	Men	-0.37	0.35	-1.06	0.29	-1.063	0.319
	Women	-0.80	0.39	-2.08	0.04	-1.560	-0.042
*θ*_*X→M1*_ *θ*_*M1→Y*_							
21–25 yrs. age	Men	-0.93	0.26	-	-	-1.457	-0.437
	Women	-1.20	0.29	-	-	-1.806	-0.665
26–34 yrs. age	Men	-0.63	0.28	-	-	-1.252	-0.132
	Women	-0.90	0.31	-	-	-1.557	-0.322
*θ*_*X→M2*_ *θ*_*M2→Y*_							
21–25 yrs. age	Men	-0.43	0.25	-	-	-0.932	0.036
	Women	0.17	0.26	-	-	-0.303	0.704
26–34 yrs. age	Men	-0.68	0.28	-	-	-1.202	-0.078
	Women	-0.08	0.30	-	-	-0.661	0.556

*Note*. 95% BCa CI = Bias-corrected and accelerated bootstrap confidence interval (CI) based on 10,000 bootstrap resamples; LL = lower limit, UL = upper limit.

**Fig 4:**
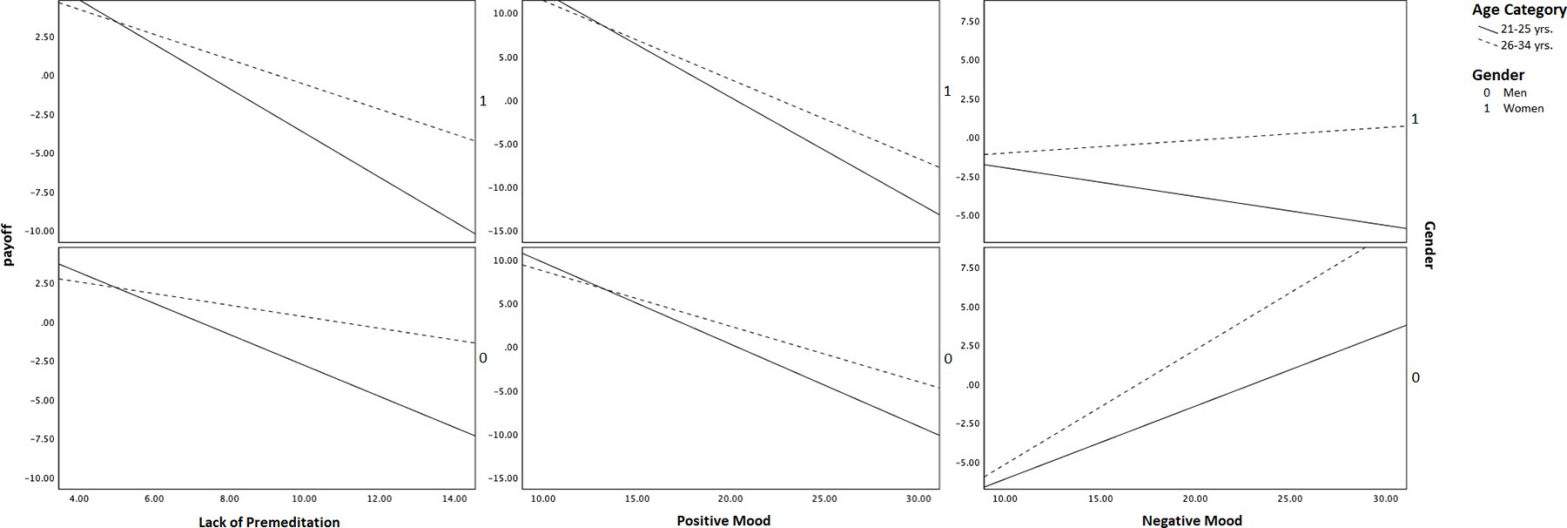
Conditional relationship in mediation-moderation model 2.

The conditional effect summary for Model 2 suggests that the 21–25 years age category significantly moderates the direct relationship between premeditation and payoff in both genders and the 26–34 years age category in women (Table 4). The indirect effect through positive mood is moderated by both age categories and genders (Table 4). The conditional indirect effect through negative mood is moderated by the 26–34 years age category in men (*b* = -.68, 95% BCa CI [-1.202, -.078]) (Table 4). The moderating conditional effects for Model 3 are visually depicted in Fig 4.

## Discussion

The present study primarily evaluates the mediating role of sensation seeking and lack of premeditation and the moderating role of age and gender in predicting payoff in Iowa Gambling Task (IGT) performance. IGT is a paradigm to assess an individual’s decision-making ability with associated risk. The ’payoff’ score describes the preference for advantageous decks (with lower rewards and lower losses) against disadvantageous decks (with greater rewards and losses), ultimately leading to the overall gain. Hence, a positive payoff score indicates beneficial decision-making, whereas a negative score indicates detrimental decision-making.

The results show that lack of premeditation, sensation-seeking, and positive mood are significantly negatively correlated with IGT payoff, while age category and negative mood are positively correlated. Literature provides evidence for decision-making ability as an age-dependent construct that varies as an individual grows [32, 33]. During adolescence, positive development in cognitive processes is critical for molding decision-making ability in a constructive way [33].

Concerning IGT performance, the literature suggests the preference for disadvantageous options during the 20s [32]. However, as people move up in age from 20s to 30s, this preference is witnessed to linearly transform into avoidance of risky decisions. Hooper et al. [34] witness the role of attention also to be age-dependent in the context of decision-making (IGT outcome). Hence, age is an important factor in determining decision-making ability.

For mediation and moderation analysis, it was hypothesized that sensation-seeking and lack of premeditation would predict the payoff (Hypothesis 1), and the mood (positive or negative) prior to performing the decision-making task moderates this predictive link (Hypothesis 2). Biological characteristics, including age and gender, are hypothesized to moderate direct and indirect relationships between predictors, moderators, and the outcome variable (Fig 2, Model 1, 2). The results provide evidence for acceptance of the first hypothesis, where both sensation-seeking and lack of premeditation significantly negatively predicted payoff. It is suggested that high sensation seekers and people lacking premeditation are likely to make disadvantageous decisions in a task with involved risk [11, 15]. These findings support previous studies that claim a significant relationship between sensation-seeking, premeditation, and decision-making [12, 14, 15].

The second hypothesis is partially fulfilled, where positive mood before IGT performance significantly negatively mediated the direct relationship between both the predictors and payoff (except 26–34 years old men with sensation seeking as a mediator). Interestingly, negative mood significantly positively predicted and positive mood negatively predicted the payoff in both Models. It means that both the negative and positive moods independently facilitate advantageous decision-making while considering the presence of sensation seeking and lack of premeditation. However, the results suggest that negative mood negatively mediates the relationship between sensation seeking and the payoff for 21 to 34 years old men and between lack of premeditation and payoff for 26–34 years old men.

Thus, mediation-moderation results propose that decision-making performance may get impaired by the positive mood in both men and women but by negative mood only in men, in high sensation seekers, and people who lack premeditation skills via indirect pathways. In contrast, a negative mood can directly facilitate decision-making in individuals with an average level of sensation-seeking and premeditation skills. Combinedly, these findings suggest that both men and women between 21 to 25 years of age, as well as men with 26–34 years of age, are more prone to disadvantageous decisions, especially if they are high sensation-seekers, lack premeditation, and are in an elevated positive mood before making decisions.

Existing literature lacks studies that explored the moderating role of affect. The studies evaluating the relationship between affect/mood and decision-making suggest that unpleasant moods lead to beneficial choices [5, 18]. However, this relationship is not proposed to be straightforward. Chung et al. [18] reported that affect is context-dependent, and different decision-making skills are adapted to different affective states and situations. The present findings support this hypothesis while providing evidence for the significant role of positive and negative affect in mediating the relationships between predictors and decision-making that operate through different age categories and genders differently. Hence, the findings add to the existing corpus of research on risky decision-making and propose further exploration to have a better understanding of these complex relationships.

## Limitations

The study was conducted on apparently healthy college students encompassing emerging and young adults. Hence, the findings may/may not be applicable to adolescents, the elderly, and clinical populations. Though the sample includes a healthy population and adults, there is a scope for extending the present study to other age groups and clinical samples.

## Conclusion

The current study explored how sensation seeking, lack of premeditation, affect, age, gender, and risky decision-making are interlinked. Evidence supports the direct role of heightened sensation seeking and lack of premeditation in disadvantageous decision-making within a risk context. Moreover, this relationship has been influenced by positive and negative moods differently in different age groups and genders. The risky decision-making is also witnessed to decrease as one move towards the 30s of their age. The mediation-moderation findings emphasize the significance of premeditative and emotional regulation training in countering various negative behavioral consequences. It also emphasizes the need to monitor younger age groups with high sensation-seeking since they are more prone to risky activities such as gambling.
